# High-resolution deposition of conductive and insulating materials at micrometer scale on complex substrates

**DOI:** 10.1038/s41598-022-13352-5

**Published:** 2022-06-04

**Authors:** Mateusz Łysień, Łukasz Witczak, Aneta Wiatrowska, Karolina Fiączyk, Jolanta Gadzalińska, Ludovic Schneider, Wiesław Stręk, Marcin Karpiński, Łukasz Kosior, Filip Granek, Piotr Kowalczewski

**Affiliations:** 1 XTPL SA, Stabłowicka 147, 54-066 Wrocław, Poland; 2 Institute of Low Temperature and Structure Research, Polish Academy of Sciences, Okólna 2, 50-422 Wrocław, Poland

**Keywords:** Electrical and electronic engineering, Electronic devices, Nanoparticle synthesis, Organic LEDs, Displays

## Abstract

Additive manufacturing transforms the landscape of modern microelectronics. Recent years have witnessed significant progress in the fabrication of 2D planar structures and free-standing 3D architectures. In this work, we present a much-needed intermediary approach: we introduce the Ultra-Precise Deposition (UPD) technology, a versatile platform for material deposition at micrometer scale on complex substrates. The versality of this approach is related to three aspects: material to be deposited (conductive or insulating), shape of the printed structures (lines, dots, arbitrary shapes), as well as type and shape of the substrate (rigid, flexible, hydrophilic, hydrophobic, substrates with pre-existing features). The process is based on the direct, maskless deposition of high-viscosity materials using narrow printing nozzles with the internal diameter in the range from 0.5 to 10 µm. For conductive structures we developed highly concentrated non-Newtonian pastes based on silver, copper, or gold nanoparticles. In this case, the feature size of the printed structures is in the range from 1 to 10 µm and their electrical conductivity is up to 40% of the bulk value, which is the record conductivity for metallic structures printed with spatial resolution below 10 µm. This result is the effect of the synergy between the printing process itself, formulation of the paste, and the proper sintering of the printed structures. We demonstrate a pathway to print such fine structures on complex substrates. We argue that this versatile and stable process paves the way for a widespread use of additive manufacturing for microfabrication.

## Introduction

Additive manufacturing provides a versatile platform for prototyping and fabrication of next-generation microelectronic devices, including displays, IoT sensors, biomedical devices, batteries, solar cells, and MEMS devices^1–7^. Significant progress in printing techniques observed in recent years was related to the fabrication of 2D planar structures and free-standing 3D architectures^8–11^. In this regard, particularly interesting are methods of direct writing^12,13^ that allow to obtain printed features in micrometer range without the use of electric field, which could be potentially dangerous to fragile electronic components on the substrate. Missing part in this picture is an intermediary technology, which would allow the printing of fine conductive and insulating structures on complex substrates. Here, by the complexity of the substrate we mean both the topography and different material properties. The printed features should closely follow the profile of the substrate to facilitate subsequent encapsulation and ensure mechanical stability. This is of great importance from the practical point of view, when thinking about using additive manufacturing technologies in the fabrication or defect-repair of modern microelectronic devices. Example applications include open-defect repair in OLED displays^14–16^ or making electrical interconnections in microLED (*µ*LED) arrays^17,18^.

The above requirements pose a number of challenges to the printing process and printing system, e.g., the need to precisely position the printing nozzle. For metallic inks, the printed structures should be characterized not only by a good electrical conductivity, but also by the adhesion to the substrate (including non-planar substrates and different materials). These two requirements are often contradictory when designing the ink. Obtaining high electrical conductivity of structures printed in micrometer range is also difficult because of scale effects and the mechanism of electrical conductivity in such fine structures.

In this contribution we demonstrate Ultra-Precise Deposition (UPD), a versatile approach to print micrometric conductive and non-conductive structures on a wide variety of complex substrates, both rigid and flexible. For conductive structures, UPD allows maskless deposition of highly-concentrated silver, copper, and gold pastes, with up to 85 wt% of solid content. The printed feature size is typically from 1 to 10 µm, and the maximum electrical conductivity obtained in this range is around 40% of the bulk value for the line with the width of 2 µm. The resulting printed structures can be bent and are uniform regardless of the wetting properties of the substrates.

Existing technologies are usually limited to certain classes of materials. For example, electrohydrodynamic (EHD) printing is based on electrodynamic interactions, and therefore a conductive ink is required. Dielectrics are difficult to jet, especially in the continuous jetting mode. One can increase conductivity by adding, for example, ionic salts or acids, but in general EHD is compatible with polar solvents, which are more jetable^19^. For inkjet printing, the limitation is the viscosity, typically in the range from 1 to 30 cP^20^. Our approach is more versatile: the process is based on a direct, maskless deposition of conductive and non-conductive materials. The challenge in this regard is to design the ink that allows to achieve the required printing resolution, but also ensures flawless printing over a long period of time, which is a necessary condition for industrial applications. For conductive materials we developed highly concentrated non-Newtonian pastes based on silver, copper, or gold nanoparticles. For printing of non-conductive structures, we used commercially available materials.

**Figure 1:**
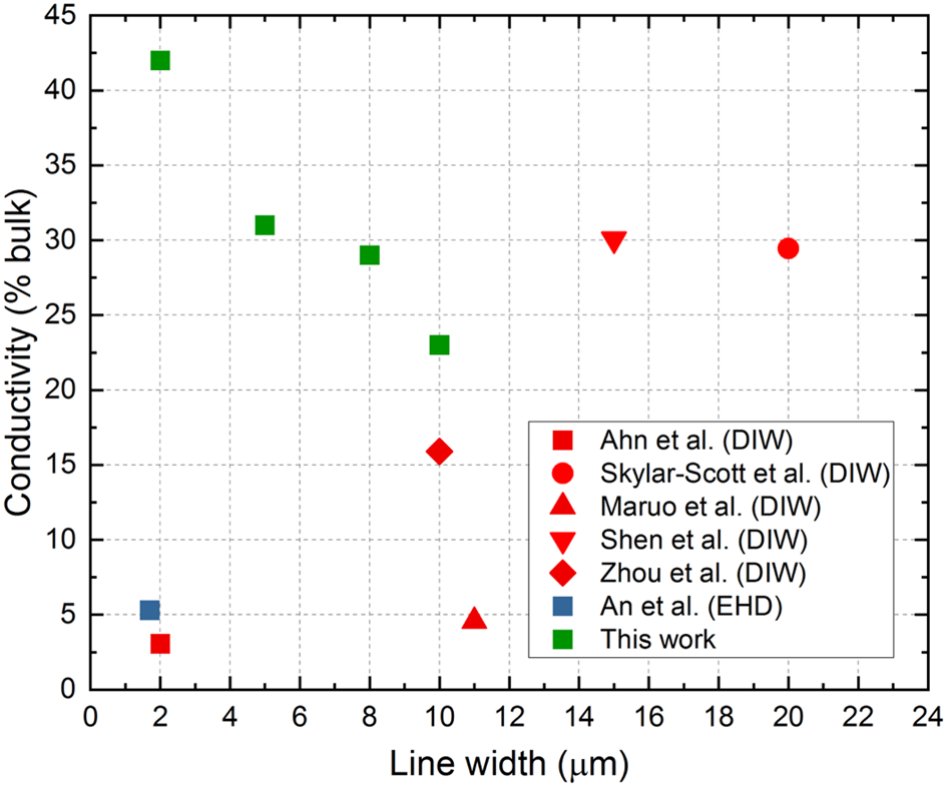
Conductivity of silver lines in terms of the percent of bulk conductivity of silver, obtained for lines with the width below 20 µm, fabricated using various additive manufacturing techniques: Electrohydrodynamic (EHD) printing, Direct Ink Writing (DIW), and Ultra-Precise Deposition (this work). Reference data were taken from^11–13,21–23^.

In Fig. 1 we show conductivity of silver lines in terms of the percent of bulk conductivity of silver, obtained for lines with the width below 20 µm, fabricated using various additive manufacturing techniques: Electrohydrodynamic (EHD) printing, Direct Ink Writing (DIW), and Ultra-Precise Deposition^11–13,21–23^. In this work, we will present the means to achieve record conductivity for micrometer-size lines fabricated using additive manufacturing technique.

These results are the effect of the synergy between: (1) paste formulation; (2) preparation of the printing nozzle; (3) precise positioning of the nozzle with respect to the substrate; (4) sintering of the printed structures to ensure proper conductivity.

We demonstrate a pathway to print such fine structures on complex substrates, discussing the process itself, its capabilities, and potential applications. In “Ultra-precise deposition” section we describe the working principle of the Ultra-Precise Deposition and the means to control the process. “Inks and pastes” section discusses different materials that can be used with the UPD approach: silver, copper, and gold pastes developed in-house, as well as commercial insulating materials. In this section we also discuss the formulation and characterization of the silver paste, which allowed us to obtain majority of the results presented in this work. “Results” section shows the key capabilities and potential applications of the UPD process. Using a number of examples, we want to address common challenges in modern flexible and printed electronics: high-resolution printing of conductive structures on rigid and flexible substrates, making electrical interconnections that require printing on steps much higher than the line width, depositing microdots (from conductive and insulating materials). We also give more specific application examples: array of source/drain structures for printed flat panel display, OLED defect repair (an example of printing on a complex substrate), and LED matrix powered by printed silver lines. Finally, “Conclusions” section concludes this work.

## Ultra-precise deposition

### Working principle of the deposition process

**Figure 2:**
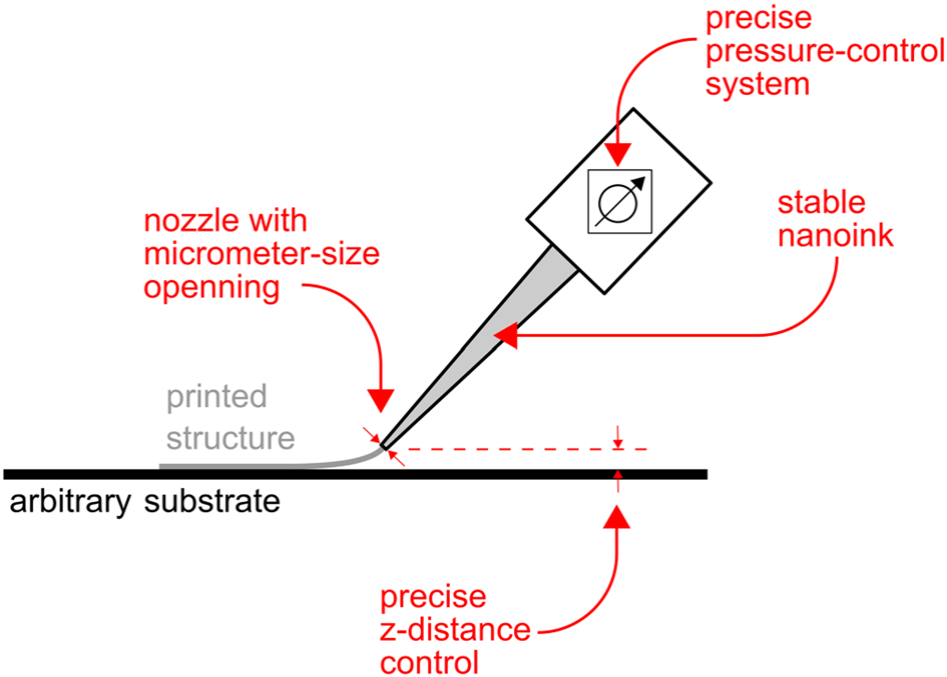
Sketch demonstrating Ultra-Precise Deposition (UPD) approach. The key feature of this technology is that high-viscosity paste (85 wt% of solid content) is extruded using a narrow printing nozzle (0.5–10 µm), which gives 1–10 µm feature size with an excellent aspect ratio in a single pass. This is achieved by a proper interplay between the paste properties, the process parameters, and the nozzle design. Finally, no electric field is required.

The unique operating range for the UPD technology, compared to other printed electronics techniques, is defined by the combination of pastes with high viscosity and fine printed features. This can be achieved by a simultaneous optimization of the paste, the process parameters, and the printing nozzle. In Fig. 2 we sketch the working principle of the UPD process: highly-concentrated paste is extruded from the printing nozzle and directly deposited on the substrate. The internal diameter of the printing nozzle is in the range from 0.5 to 10 µm, and the tip of the nozzle can be either in direct contact with the substrate or positioned at a certain height above the substrate, up to tens of micrometers. The nozzle position is one of the parameters to control the process and the positioning is performed using a high-resolution vision system. To obtain uniform structures, the nozzle substrate distance must be kept constant for the time of printing. The process is governed by pressure, and thus the precise pressure-control system is a vital part of the setup. After turning on the pressure, the nozzle starts to move in the desired direction (x, y, or z). The time delay between turning on the pressure and the start of the nozzle movement is adjustable. If this time is too long, the material may spill and leave an artifact at the beginning of the printed structure. On the other hand, if this time is too short, it may result in printout discontinuities. The optimal delay time is related to the inertia of the pneumatic system and depends on the nozzle opening, material density, and pressure. Finally, the value of the applied pressure has to be above a certain threshold that allows extruding the paste from the nozzle. This threshold value depends mostly on the nozzle opening and properties of the paste. To finish the printing process, the pressure is reduced below the threshold value.

**Figure 3:**
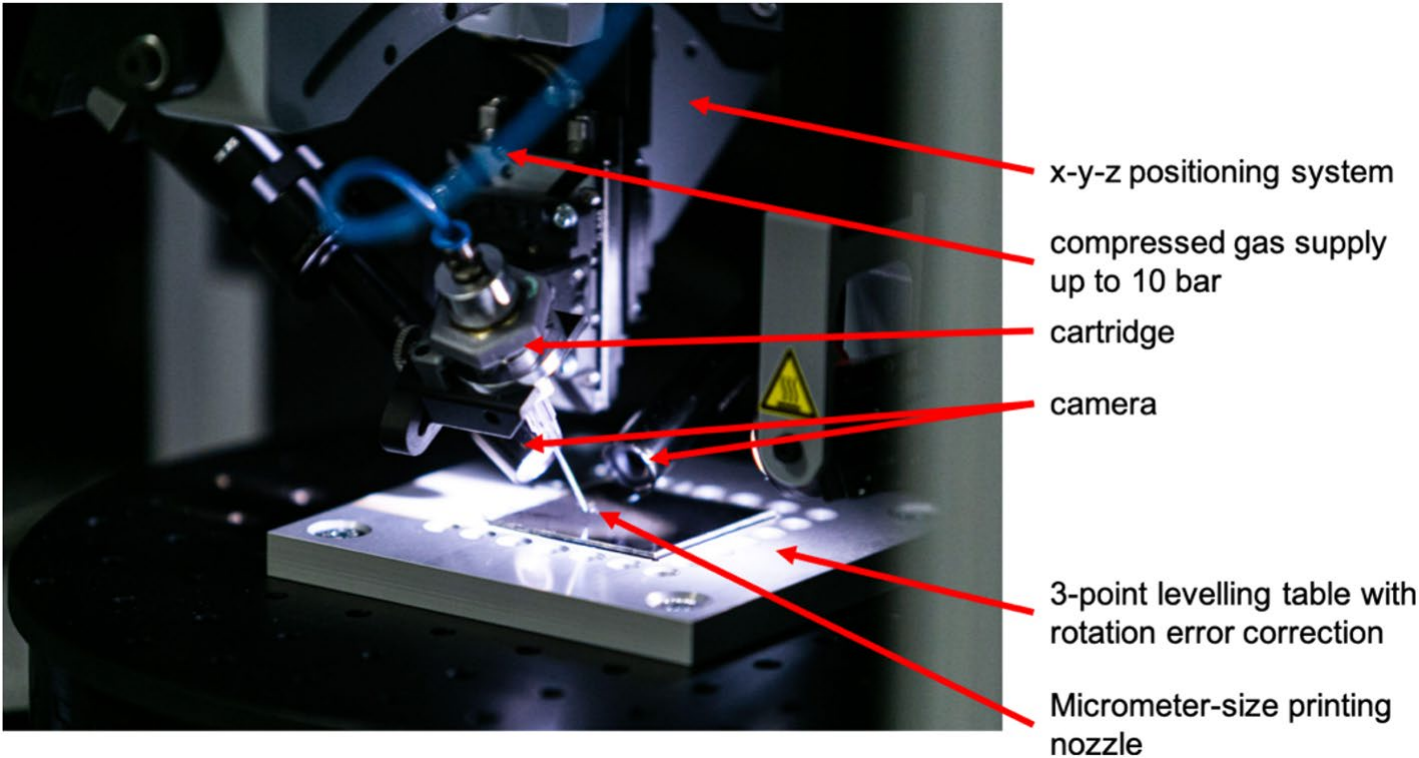
Photo of the printing system implementing the UPD technology. The sample is placed on a 3-point levelling table with rotation error correction. The printhead is positioned using 3-axes positioning system and vision algorithm.

In Fig. 3 we show a photo of the printing system implementing the UPD technology. The main elements of the system are: micrometer-size printing nozzle, 3-point levelling table with rotation error correction, positioning system and compressed gas supply. The positioning system consists of XY motor with the movement accuracy of 2 µm and Z motor with the movement accuracy of 0.5 µm. The compressed gas supply gives the maximum pressure of 10 bar. The maximum printing speed with this setup is 10 mm/s.

Due to the proper design of the paste, it is stable inside the nozzle and can be extruded through such a narrow opening. The viscosity of the pastes can reach the value of 2,400,000 cP. Yet, the printing process is possible because of the non-Newtonian nature of these fluids: the effective viscosity of the paste at the tip of the nozzle is of the order of tens of centipoise, due to shear thinning. Optimization of physico-chemical properties of the pastes is described in “Inks and pastes” section.

The printed structures are characterized by feature size in the range from 1 to 10 µm and remain uniform regardless of the wetting properties of the substrate: the moment the paste is extruded from the nozzle, its viscosity returns to the stationary value. Therefore, it is possible to print on materials with very different wetting properties, such as oxides (e.g., SiO_2_), nitrides (e.g., SiN_x_), metals, glass, and foils (e.g., PI, Kapton), as well as to print on junctions (metal/semiconductor/insulator) and to cover vertical steps.

**Figure 4:**
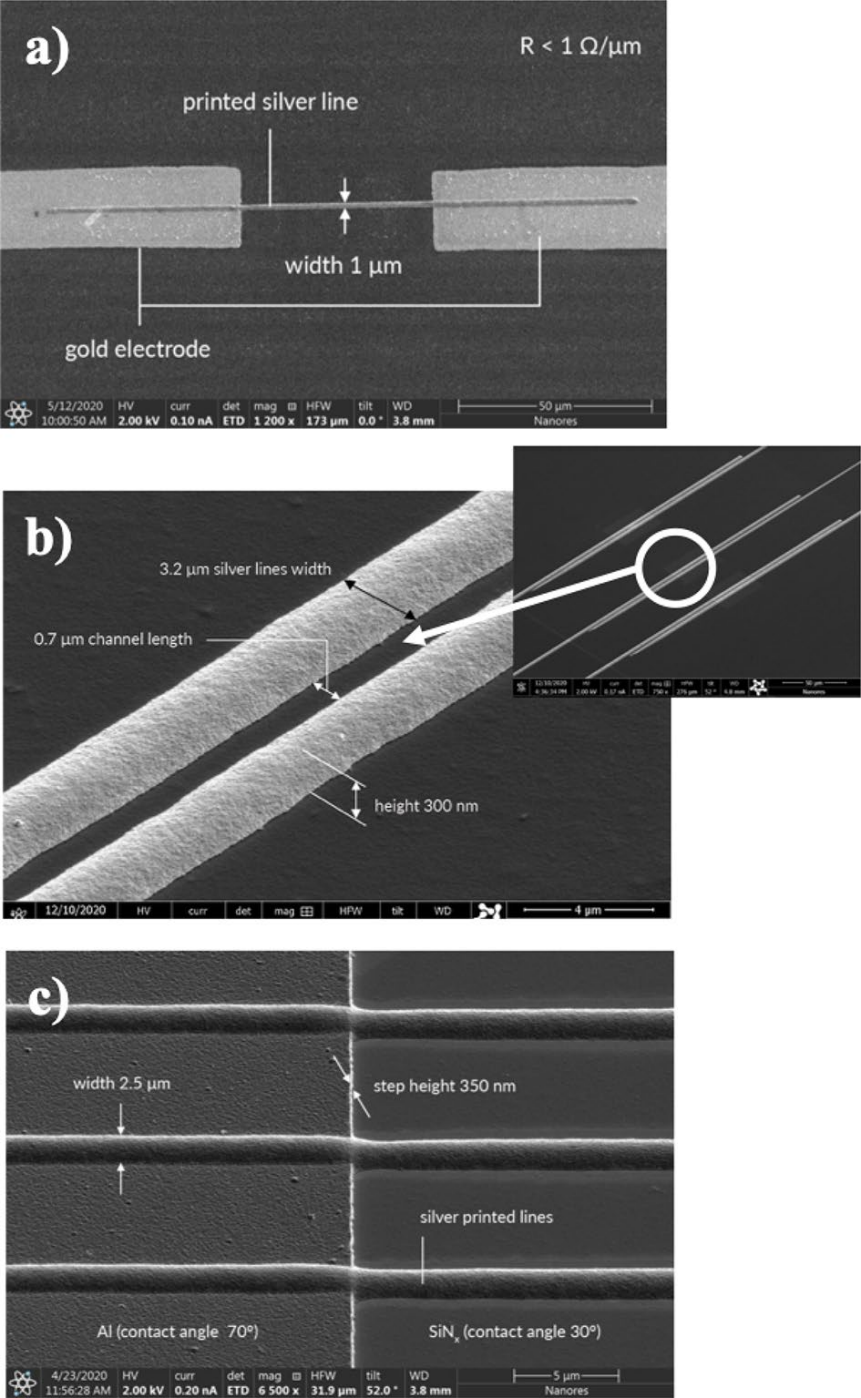
(**a**) Silver line with the width of 1 µm printed between two gold electrodes on glass. The electrical resistance of the line is below 1 Ω/µm; (**b**) silver lines with the width of 3 µm printed on a PEN foil. The line width is 3.2 µm and the interline distance is 0.7 µm. Note that the interline distance is kept constant and the printed lines are clearly separated. Distant view of the sample is shown in the inset; (**c**) hybrid substrates composed of materials characterized by different wetting properties: aluminum (Al) layer deposited on a PECVD silicon nitride (SiN_x_) with a vertical step of 0.35 μm.

To give a simple example of high-resolution printing of conductive features at micron scale: In Fig. 4a we demonstrate silver line with the width of 1 µm printed between two gold electrodes. The electrical resistance of the line is below 1 Ω/µm. UPD provides means for high-resolution printing of conductive structures, with the resolution defined both as the size of the printed features, as well as the distance between them. In Fig. 4b we show an example set of silver lines printed on a PEN foil. The line width is 3.2 µm and the interline distance is 0.7 µm (distant view of the sample can be seen in the inset). It is important to notice that the lines are clearly separated: the interline distance is kept constant and there are no points of contact, which would result in a short circuit.

Hybrid substrates, composed of materials characterized by different wetting properties, are common in novel electronic devices. An example of printing on such a hybrid substrate is shown in Fig. 4c. The substrate consists of aluminum (Al) layer deposited on a PECVD silicon nitride (SiN_x_) with a vertical step of 0.35 μm. The printed silver lines are characterized by the width of 2 μm and the height of 350 nm. Despite different contact angles of Al and SiN_x_, the printed structures are uniform, also at the interface of both materials. Moreover, the lines cover the step without discontinuities and the proper sintering ensures a full adhesion to both materials.

### Control of the process

The key parameters to control the printing process are: internal opening of the nozzle, process pressure, and printing speed. These three parameters determine the mass flow, i.e., the amount of material deposited on the substrate per unit area, which in turn impacts the width and height of the printed structures. As discussed above, another parameter to control the process is time delay between turning on the pressure and movement of the nozzle. This delay impacts the homogeneity and continuity of the printed structures. The distance between the tip of the nozzle and the substrate also impacts the width and height of the printed structures.

The nozzle can be positioned at various angles with respect to the substrate. The practical range is from 90° (nozzle positioned perpendicularly to the substrate) to 40°. Positioning the nozzle at 90° facilitates the printing in *x* or *y* direction. However, this position is not suitable for printing with higher printing speeds (more than approximately 0.2 mm/s). Positioning the nozzle at 50°–60°. makes it more mechanically stable. In this case, printing in the direction in which the nozzle is tilted is preferred. Yet, for the tilted nozzle, the process parameters have to be tuned, depending on the direction of printing. The reason is that the internal opening of the nozzle is positioned differently with respect to the substrate, depending on the printing direction. For each nominal line width, we calculate standard deviation of width and height. For example, for lines with the width of 5 µm, standard deviation of width is 0.24 µm and standard deviation of height is 0.02 µm. For other line widths (and nozzle sizes) the values of standard deviation are similar in relative terms.

**Figure 5:**
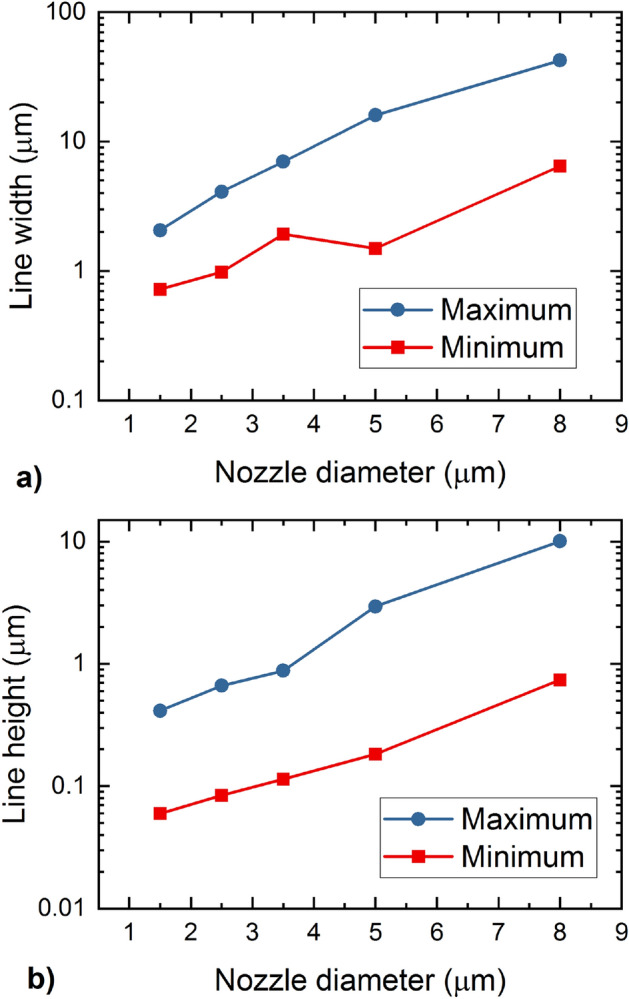
Line width (**a**) and height (**b**) with respect to the nozzle opening. The nozzle was tilted at 50 deg with respect to the substrate.

In Fig. 5 we demonstrate the ranges of width and height of the printed lines, which are possible to obtain with different openings of the nozzle. From Fig. 5a it can be seen that the narrowest lines are obtained for the smallest nozzle (1.5 µm). The line width in this case is in the range from approximately 0.7–2 µm. Increasing the nozzle opening increases the line width, as well as the range. For the largest nozzle (8 µm), the line width is in the range from 6.5 µm to 40 µm. In Fig. 5b we show similar trends for the line height. The thinnest lines are printed using the smallest nozzle (1.5 µm), and the line height ranges from approximately 0.06–0.4 µm. Increasing the opening increases the line height, as well as the range. For the largest nozzle (8 µm), the height is in the range from approximately 0.7–10 µm.

**Figure 6:**
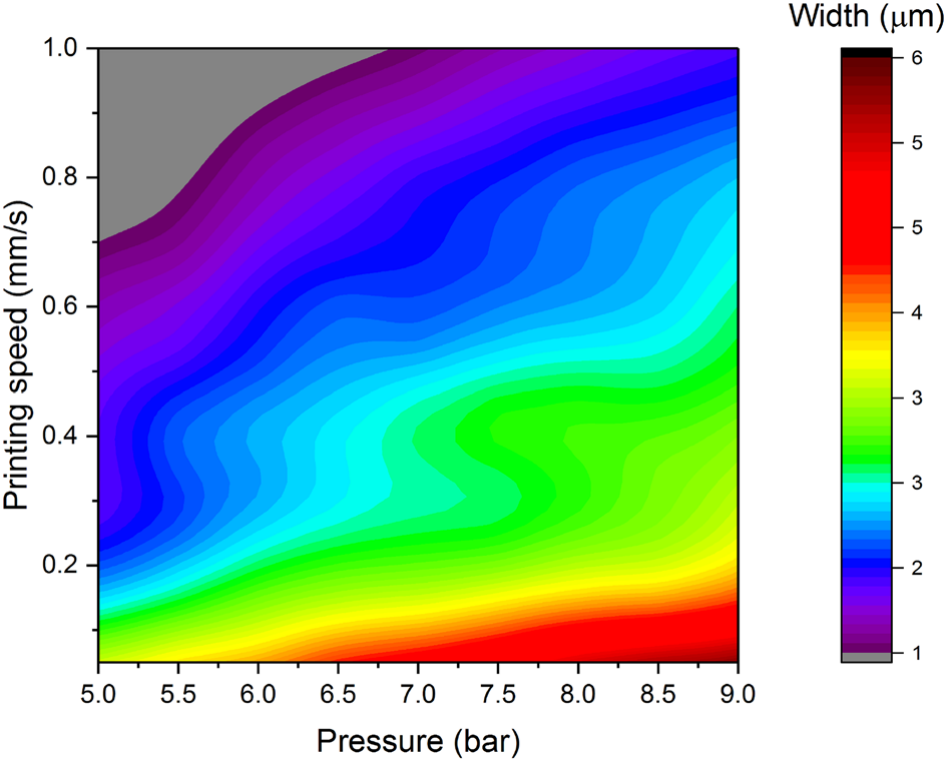
Line width as a function of the printing pressure and printing speed for the nozzle with the opening of 3.5 µm.

In Fig. 6 we show how the width of the printed lines depends on the printing pressure and speed for the nozzle with the external opening of 3.5 µm. It can be seen that the widest lines (> 5 µm) can be achieved by the combination of low printing speed (below 0.1 mm/s) and high printing pressure (more than 8 bar). For the narrowest lines, the situation is opposite. Higher printing speed (> 0.4 mm/s) and low printing pressure (< 8 bar) give printed structure as narrow as 1 µm. Yet, these parameters do not impact the line width in the same way. The most significant differences can be observed for low printing speed and high printing pressure. Changing the printing speed from approximately 0.05–0.4 mm/s gives more significant change in the line width, compared to changes in the high-speed region of the plot. The same behavior can be seen for high printing pressure (from 7 to 9 bar). Finally, in the gray region of the plot, for the high printing speed and low pressure, we were not able to obtain continuous lines, which sets the current limit of this technology in terms of the feature size of the printed structures.

**Figure 7:**
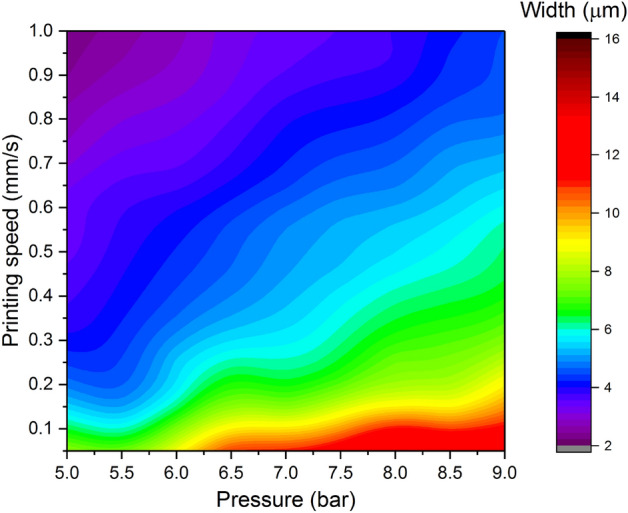
Line width as a function of the printing pressure and printing speed for the nozzle with the opening of 5 µm.

Similar trends can be observed for the nozzle with the opening of 5 µm, as shown in Fig. 7. What changes is the size of the printed structures: the line width is in the range from around 2–3 µm (printing speed > 0.8 mm/s, pressure < 4 bar) to approximately 16 µm (printing speed of 0.05 mm/s, pressure > 8 bar). Therefore, the line width is proportional to the pressure and inversely proportional to the printing speed.

## Inks and pastes

In this section we begin with the review of materials that can be used with the UPD approach. Then, we focus on the synthesis and characterization of the silver paste, which allowed us to obtain most of the results in this work. Finally, we discuss the stability of the paste, demonstrating continuous printing for 8 h, as well as on and off printing for 60 days. The stability of the printing process is a necessary condition for scaling up an additive manufacturing technology for industrial applications.

### Overview of materials

A list of example conductive and non-conductive materials printed using the UPD technique and demonstrated in this work is shown in Table 1. The conductive materials (silver, copper, and gold pastes) were developed in-house, whereas the insulating materials were obtained from commercial providers.

**Table 1:** A list of example conductive and non-conductive materials printed using the UPD technique and considered in this work.

Type	Material	Solid content (wt. %)	Average NP size (nm)	Viscosity (cP)	Typical printed feature size (μm)	Conductivity of printed structures (% of bulk)
Conductive (developed in-house)	Ag nanopaste (CL85)	85 ± 2	35–50	> 10^5^	1–10	Up to 40
	Cu nanopaste	85 ± 2	50–70	> 10^5^	5–20	Up to 21
	Au nanopaste	80 ± 2	50–80	> 10^5^	8 – 20	Up to 40
Insulating (external supplier)	Photoresist AR-P 3110	N/D	N/D	12	2–20	–
	SU8	N/D	N/D	N/D	2.5–100	–

Viscosity is measured at 25 °C and shear rate = 0.2 s^–1^. Photoresist AR-P 3110 was ordered from Allresist GmbH, whereas SU8 was ordered from Sigma-Aldrich.

It can be seen that the viscosity of the materials compatible with the UPD method ranges from a few cP up to more than 10^5^ cP for non-Newtonian pastes. The smallest feature size and the highest conductivity is obtained for the silver paste. Finally, for metallic nanoparticles, the average NP size is in the range from 25 to 80 nm.

### Design and characterization of the silver paste

To achieve highly-conductive micrometer lines, the UPD method requires pastes with very specific characteristics, which makes the pastes formulation a vital part of the process. The pastes exhibit both high stability and homogeneity, which are essential to prevent the agglomeration of the nanoparticles, sedimentation, and other separation phenomenon, as well as prohibiting fluctuation in the paste properties over time. Solvents and additives play a great role in preventing these events. Drying and clogging of the pastes are rarely described in the literature, especially in the field of 3D printing, when formulations characterized by high metal concentrations are used. Although drying is very likely to occur, pastes which are used with the UPD method can go as high as 85 wt.% of solid content without clogging for long working times, while retaining their properties. Therefore, in this work we present products that are industrially viable, as they will not suspend or prolong the manufacturing process.

Narrowness and height-to-width aspect ratio of printed structures originate both from the high viscosity and in-built rheological behavior, which at the same time allow easy dispensing and facilitate shape retaining of the extruded paste. Therefore, the rheological behavior of the paste represents a crucial parameter for the UPD method. We use solvents and additives that enable good behavior of the pastes during the sintering process. This often-overlooked aspect is a key to prevent the appearance of internal and external stresses or damages, which can impact the final properties of the sintered material, and results in the loss of conductivity or even lower mechanical resistance. Adhesion promoters enable the printed lines to pass the 10 × Scotch-tape test and are employed at concentrations which minimize conductivity loss in the printed structure.

**Figure 8:**
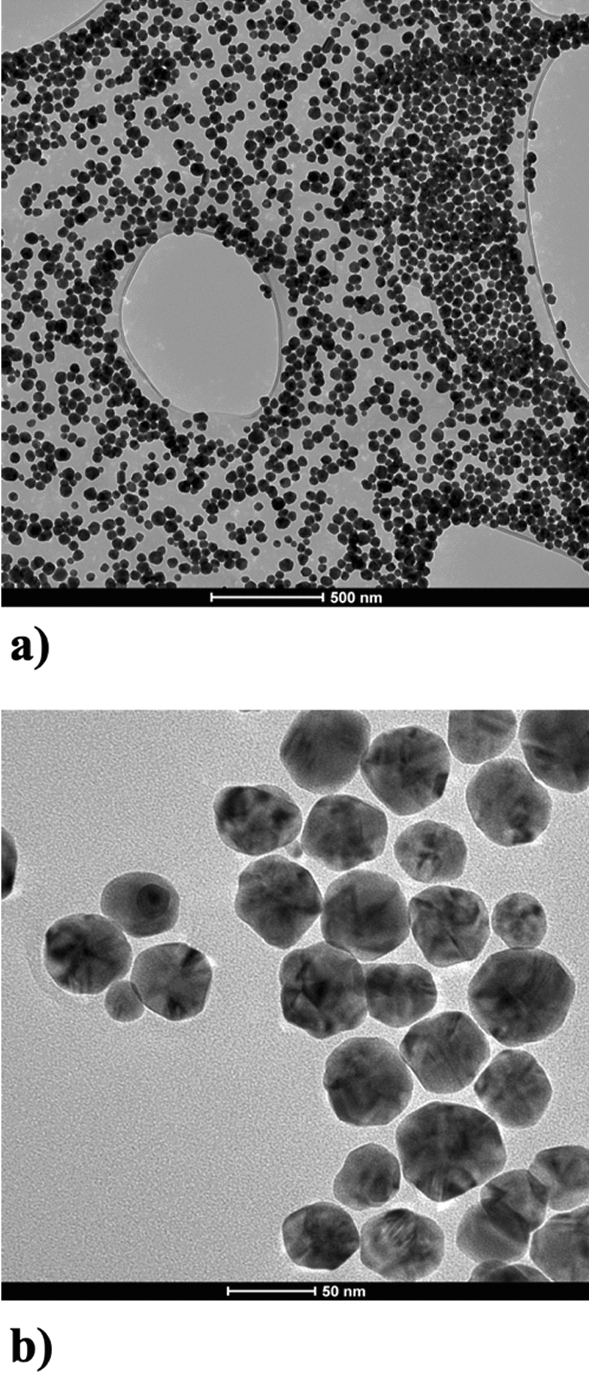
Transmission electron microscopy (TEM) images of obtained silver nanoparticles (Ag NPs).

**Figure 9:**
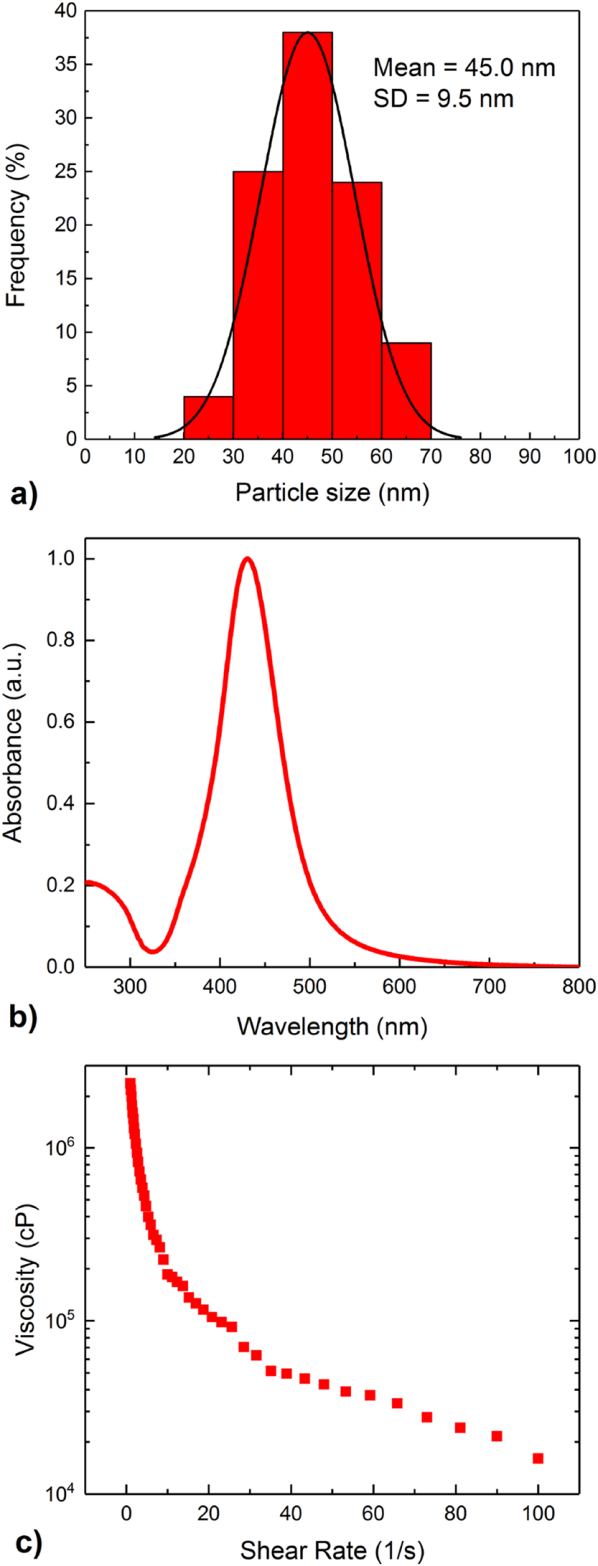
Size distribution of the silver nanoparticles, together with Gaussian fit. The mean is equal to 45 nm and the standard deviation (SD) is equal to 9.5 nm (**a**); UV–Vis spectrum of silver nanoparticle paste (85 wt% of solid content) (**b**); as well as viscosity as a function of the shear rate (**c**).

AgNPs morphology and size distribution were characterized by transmission electron microscopy (FEI Tecnai G2 X-TWIN). Homogeneous spherical nanocrystals with average size of 45 nm and narrow size distribution were observed (Figs. 8, 9a). The paste was diluted with ethylene glycol and UV–VIS spectrum was collected (Perkin Elmer Lambda 650), which showed a highly intensive and symmetrical band at *λ*_max_ = 430 nm (Fig. 9b), characteristic for surface plasmon resonance of silver nanoparticles. Final product is estimated to have a solid content of 85 wt% based on gravimetric analysis and silver concentration of 83 wt% determined by ICP/OES Thermo Scientific iCAP 7400 Duo. For rheological characterization, Anton Paar MCR 92 rheometer with plate-plate PP25 measurement system was used. The viscosity as a function of the shear rate (Fig. 9c) shows that the paste viscosity decreases dramatically from approx. 2,400,000 cP to even below 100,000 cP at higher shear rates, which indicates that the paste behaves like pseudoplastic, non-Newtonian fluid.

For conductive inks, we use nanoparticles with quasi-spherical shape and we want to achieve as narrow size distribution as possible. Such homogeneity helps to avoid clogging of the printing nozzle—for higher polydispersity nanoparticles are likely to accumulate at the walls of a microchannel and clog it^24^. Inks based on Ag nanowires are generally not compatible with this printing method. The reason is that nanowires are typically much longer than the internal diameter of the outlet of the printing nozzle^25,26^, which in turn causes the nozzle to clog.

### Stability tests

**Figure 10:**
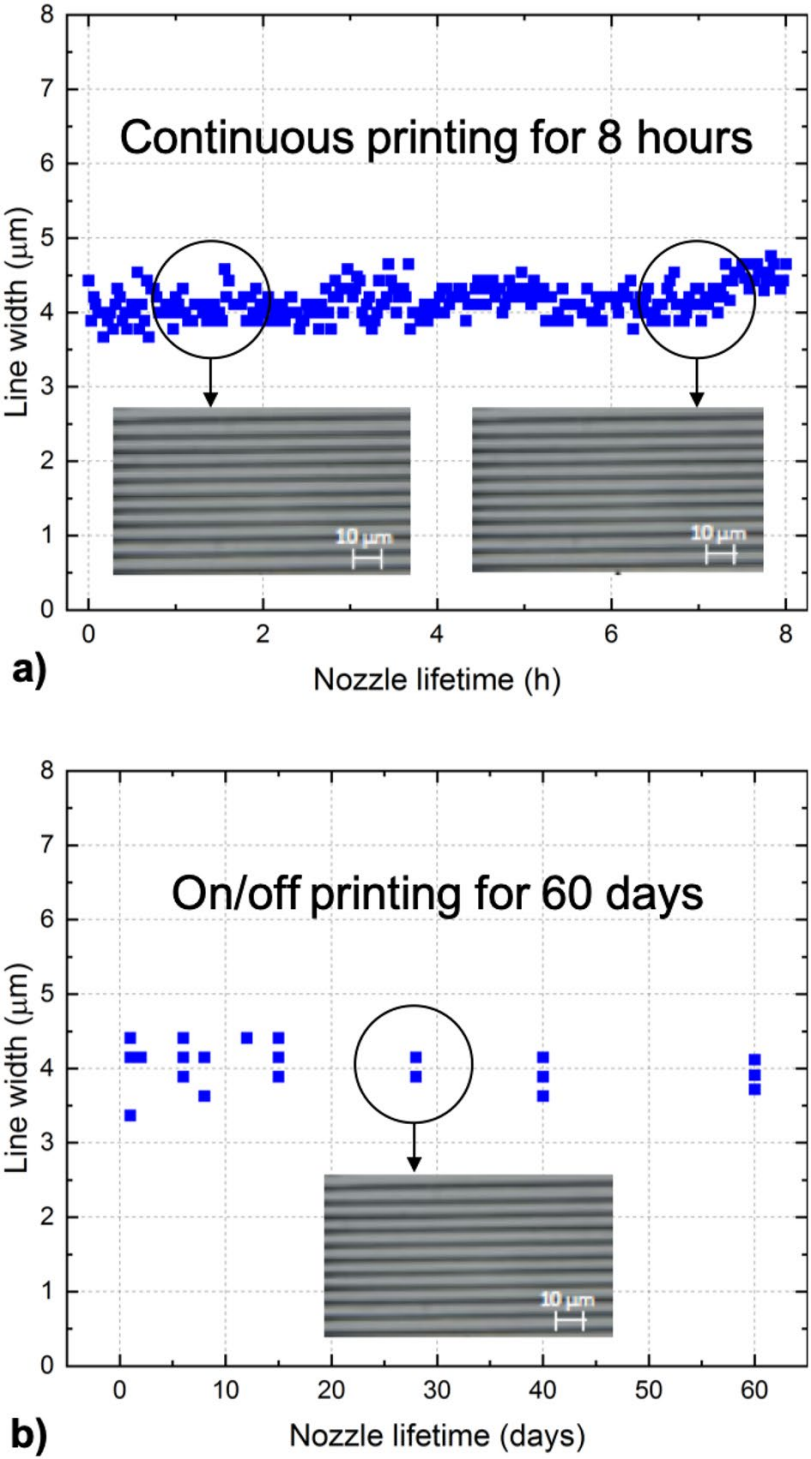
Stability tests performed for silver nanopaste and a single printing nozzle with the diameter of 2.5 µm. (**a**) continuous printing for 8 h; (**b**) on and off printing for 60 days. The scale bar in the images corresponds to 10 µm.

The key aspect of the paste is its stability in time, particularly when used with a micrometer-size nozzle. Stability is the necessary condition for scaling up the technology from laboratory to industrial applications. In Fig. 10 we show stability tests performed for silver nanopaste (85 wt% of solid content) and a single printing nozzle with the diameter of 2.5 µm. In Fig. 10a we show results obtained for continuous printing for 8 h; whereas in Fig. 10b we demonstrate results obtained for on and off printing recorded for 60 days. There was no clogging observed and standard deviation of the data points is σ = 0.21 µm for a) and σ = 0.27 µm for b).

## Results

In this section we demonstrate the key capabilities of the UPD technology as an additive manufacturing method for microfabrication. By this selection of examples, we want to demonstrate the versality of the UPD approach, in terms of the material to be printed (metallic pastes, insulating inks), shape of the printed structures, as well as type and shape of the substrate (including substrates with pre-existing features, i.e., steps).

### Conductive meshes: gold, silver, and copper

**Figure 11:**
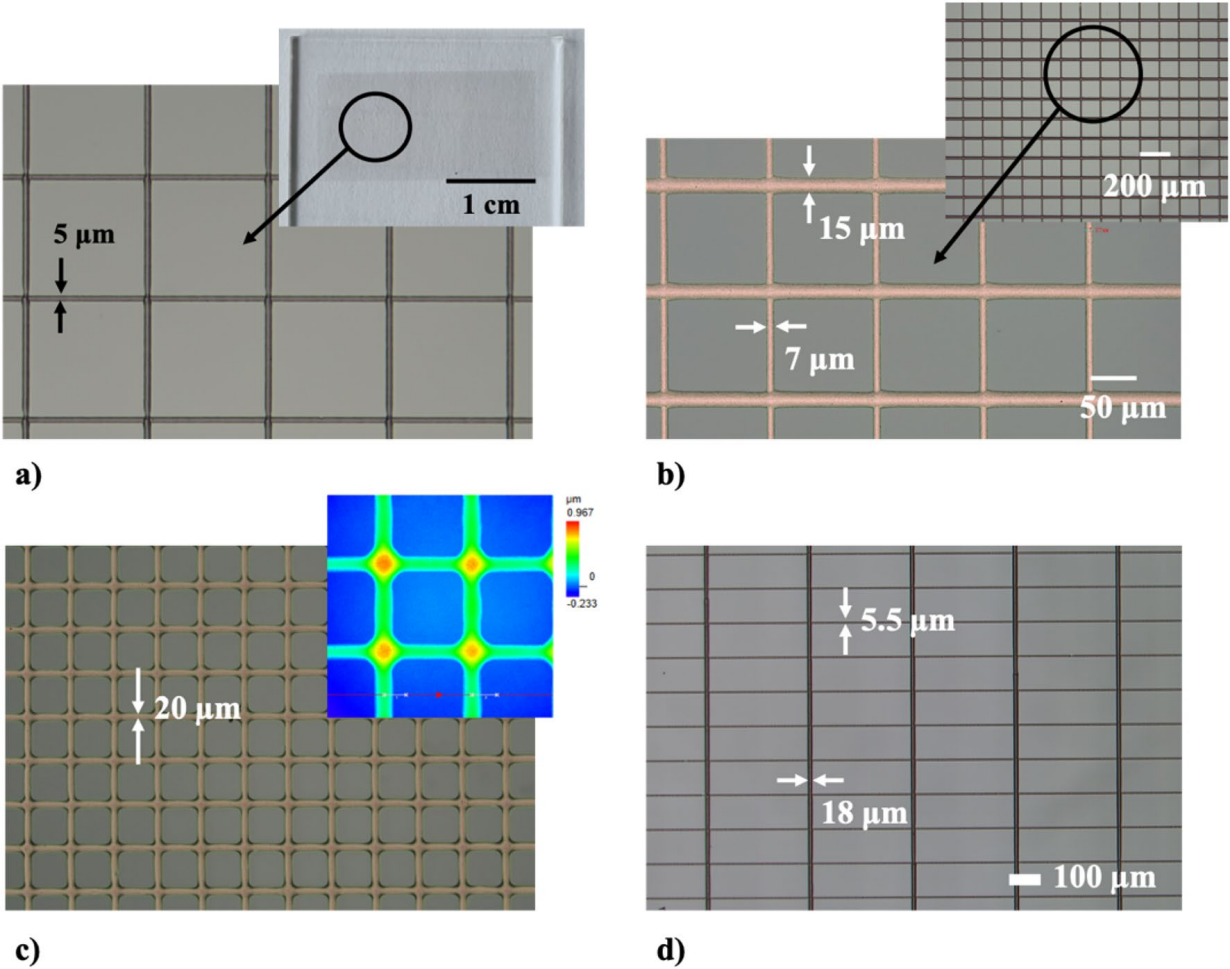
Example conductive meshes printed using highly-concentrated pastes. (**a**) Conductive mesh with 5 μm wide lines, printed using paste based on silver nanoparticles (85 wt% of solid content). The whole sample is shown in the inset; (**b**) conductive mesh printed using copper paste (80 wt% of solid content). The line width is 7 μm (horizontal direction) and 15 μm (vertical direction). The inset shows distant view of the sample; (**c**) conductive mesh printed using gold paste (80 wt% of solid content). In the inset we show a heat map corresponding to the height profile; (**d**) conductive mesh printed on glass/IZO substrate using paste based on silver nanoparticles (85 wt% of solid content). The line width is 5.5 μm (horizontal direction) and 18 μm (vertical direction).

Conductive meshes are a vital part of rigid and flexible displays, as well as e-paper, transparent displays, and smart devices. However, the choice of the conductive material depends on various design constraints, cost analysis, specific architecture, and compatibility with other components of the device. In this section we will present conductive meshes printed using pastes based on silver, copper, and gold nanoparticles. In Fig. 11a we demonstrate a conductive mesh with 5 μm wide lines printed using paste based on silver nanoparticles (85 wt% of solid content). The whole sample is shown in the inset; in Fig. 11b we show a conductive mesh printed using copper paste (80 wt% of solid content). In this design the lines have various widths: 7 μm in the horizontal direction and 15 μm in the vertical direction. The inset shows lower magnification of the sample. In Fig. 11c we present a conductive mesh printed using gold paste (80 wt% of solid content). In the inset we show a heat map corresponding to the height profile. Finally, in Fig. 11d we demonstrate conductive mesh printed on glass/IZO substrate using paste based on silver nanoparticles (85 wt% of solid content). The line width is 5.5 μm (horizontal direction) with pitch of 105 μm and 18 μm (vertical direction) with pitch of 315 μm. Such design results from a trade-off between the optical and electrical properties of the film. The mesh was sintered in 250 °C for 10 min. It allowed us to achieve sheet resistance of 1.5 Ω/□ with the transparency of 90%. Commonly used transparent conductive films (TCFs) based on indium tin oxide (ITO) are characterized by sheet resistance in the range 10–25 Ω/□ with transparency > 90%. Therefore, TCF prepared using the UPD approach gives significantly lower sheet resistance with similar transparency. Compared to TCFs prepared using other additive manufacturing techniques, transparent electrodes can be prepared using EHD printing, achieving sheet resistance of 3 Ω/□ and transparency of 96%^27^. For TCFs printed using Inkjet, one obtains lower transparency due to limited resolution of the printing process, e.g., sheet resistance of 13 Ω/□ with transparency of 81.9%; we note, however, low sintering temperature of 60 °C^28^.

### Printing on steps

**Figure 12:**
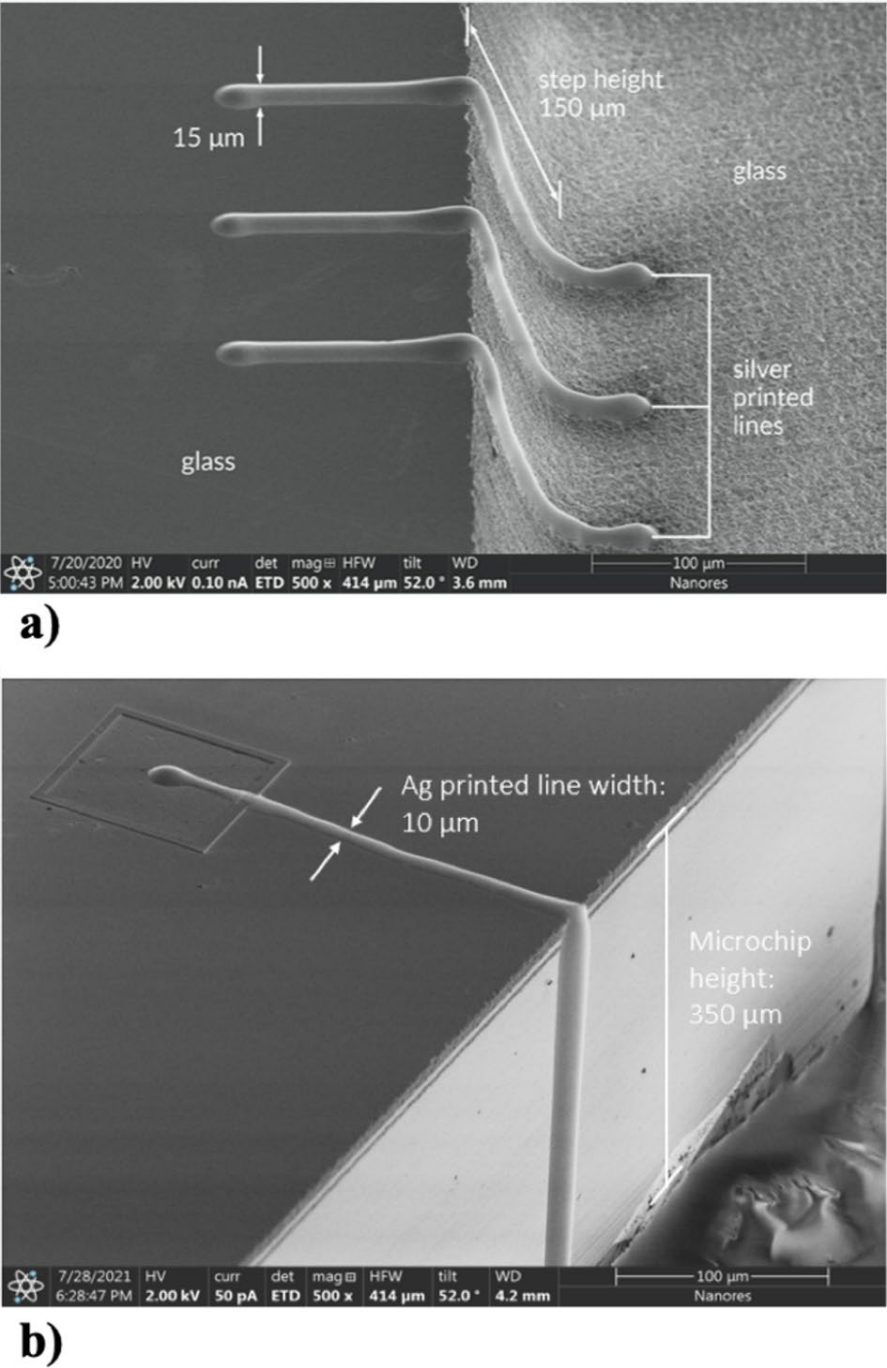
(**a**) Repeatable and continuous silver lines with a width of 15 µm printed on the step with the height of 150 µm, (**b**) 10 µm wide silver line printed on a microchip with the height of 350 µm.

UPD gives the possibility to print 3D interconnections for advanced packaging, including hybrid electronics (combining printed electronics and silicon technologies)^1,29^. In this regard, in Fig. 12 we demonstrate the capabilities of the UPD technology to print on steps with various heights. Figure 12a demonstrates repeatable and continuous silver lines with a width of 15 µm printed on the step with the height of 150 µm. Therefore, the step height is ten times the width of the lines. Figure 12b shows a 10 µm wide silver line printed on a microchip with the height of 350 µm. The range of the line widths, as well as the range of the step heights demonstrated in the above examples fully satisfies the requirements for the fabrication of interconnects in modern microelectronic devices, such as µLED arrays.

### Microdot deposition

**Figure 13:**
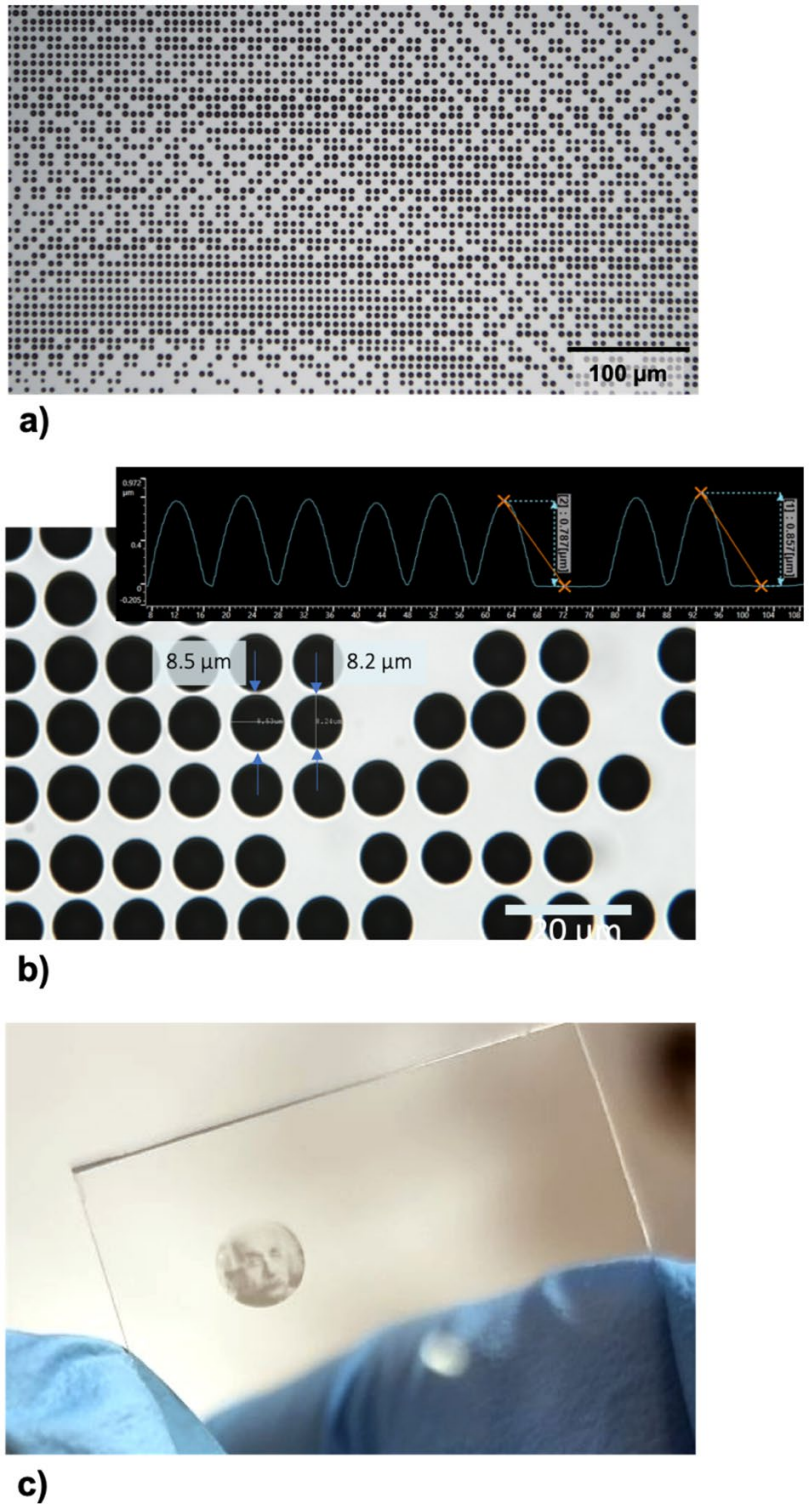
(**a**) Microdots printed using the UPD approach, the bar denotes 100 μm; (**b**) higher magnification of the sample, the bar denotes 20 μm and the inset shows cross section of the dots profile obtained using a profilometer; (**c**) an example of high-resolution picture consisting of microdots printed using the UPD approach.

Another capability of the UPD method is the possibility to print microdots. Microdots made from a conductive material can be used for making electrical contacts. In Fig. 13a we show microdots printed using the UPD approach (the bar corresponds to 100 μm), whereas in Fig. 13b we demonstrate higher magnification of the sample (the bar denotes 20 μm). In the inset of Fig. 13b there is a cross section of the dots profile obtained using a profilometer. The dots have the diameter of around 8 µm and the height of around 800 nm. One can also notice a smooth, lens-like shape of the microdots, which is desired, e.g., in the case of deposition of additional layers. Such a shape significantly reduces the risk of cracks in the subsequent layers. This is very different from microdots obtained using lithography, which generally have a rectangular shape. Finally, Fig. 13c we show an example of high-resolution picture consisting of microdots printed using the UPD approach.

The key advantages of using the UPD method to print microdots are: (1) high resolution, diameter below 10 µm; (2) high repeatability and printing stability; (3) high aspect ratio (800 nm height to 8 µm diameter); (4) the ability to cover large areas. These results also demonstrate the ability to deposit a precisely defined amount of material.

### Deposition of insulating materials

**Figure 14:**
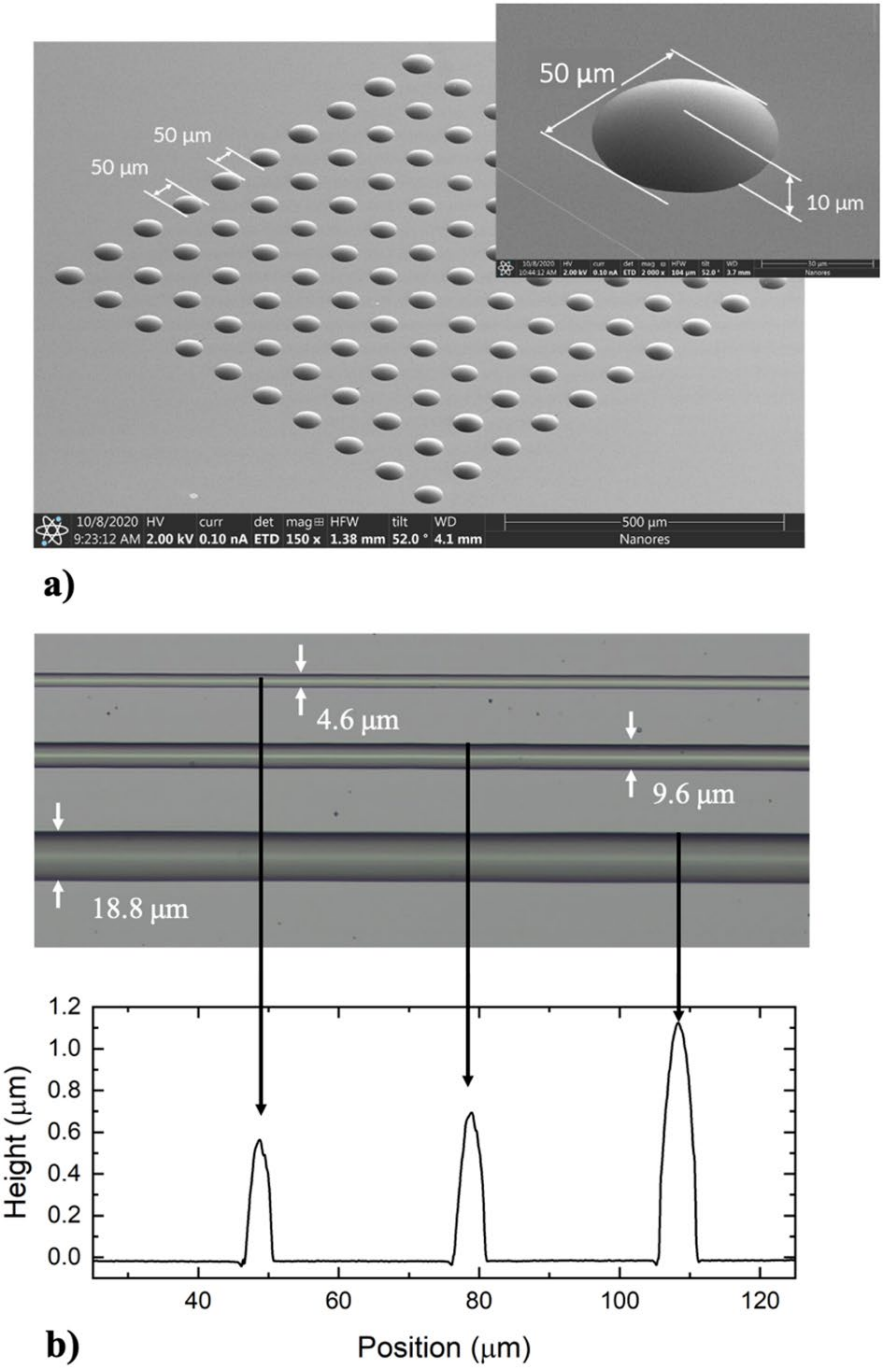
(**a**) Array of microdots printed with photoresist AR P 3110, characterized by the viscosity of 12 cP; (**b**) Set of lines printed using SU-8 photoresist together with the height profiles.

Up to now we have discussed printing using high-viscosity conductive materials developed in-house. In this section we focus on insulating materials obtained from external suppliers. In Fig. 14a we show an array of microdots printed with photoresist AR-P 3110 (obtained from: Allresist GmbH). This material is characterized by the viscosity of 12 cP, therefore orders of magnitude lower than in the case of the conductive pastes. The distance between the printed dots is equal to 50 μm, the dot diameter is equal to 13 μm, and the dot height is equal to 10 μm. The microdots are characterized by parabolic shape, which may, for example, simplifying the deposition of subsequent layers without the risk of cracks.

As another example, in Fig. 14b we show a set of lines printed using SU-8 photoresist provided by Sigma-Aldrich. Below the image of the lines there are the height profiles. We demonstrate lines with the width raging from 4.6 μm to nearly 19 μm. As in the previous examples, the line width depends on the nozzle opening, printing speed, and applied pressure.

### Open-defect repair in OLED arrays

Displays based on organic light-emitting diodes (OLEDs) have significant advantages over other display technologies^14–16^. This includes superior image quality and lower power consumption. Yet, to achieve a dominant position in the market, OLED displays require manufacturing technologies that offer high throughput, precision, and low cost at the same time. Yield management is one of the biggest challenges and it strongly affects the overall costs. In this regard, the ability to repair open defects is in high demand for OLED manufacturers^17^.

Open defects are defined as a local lack of conductive material in an OLED TFT array. Such defects may appear at the production stage and usually result in the product rejection. The problem is becoming even more significant in the case of large-area displays and ongoing miniaturization of display components with the aim of increasing the display resolution.

Current repair methods include^30–34^: Electrohydrodynamic (EHD) printing, Laser Chemical Vapour Deposition (LCVD), and Laser-Induced Forward Transfer (LIFT). The main disadvantages of these technologies are limited throughput and cost. Moreover, EHD can damage active electronic systems in integrated circuits (due to electrostatic discharge defects); LCVD, and LIFT provide only a limited possibility to obtain paths with a width below 10 µm, and LCVD uses toxic gases. For microdispenser technique for disconnection repair^12^, printing lines with the line width below 10 µm is only possible with a mask film.

An OLED display array is a complex substrate, both regarding the topography and composition of the materials. To repair a defect, it is necessary to properly position the printing nozzle and ensure that the printed connection is uniform and continuous, regardless of the shape of the substrate and its wetting properties.

**Figure 15:**
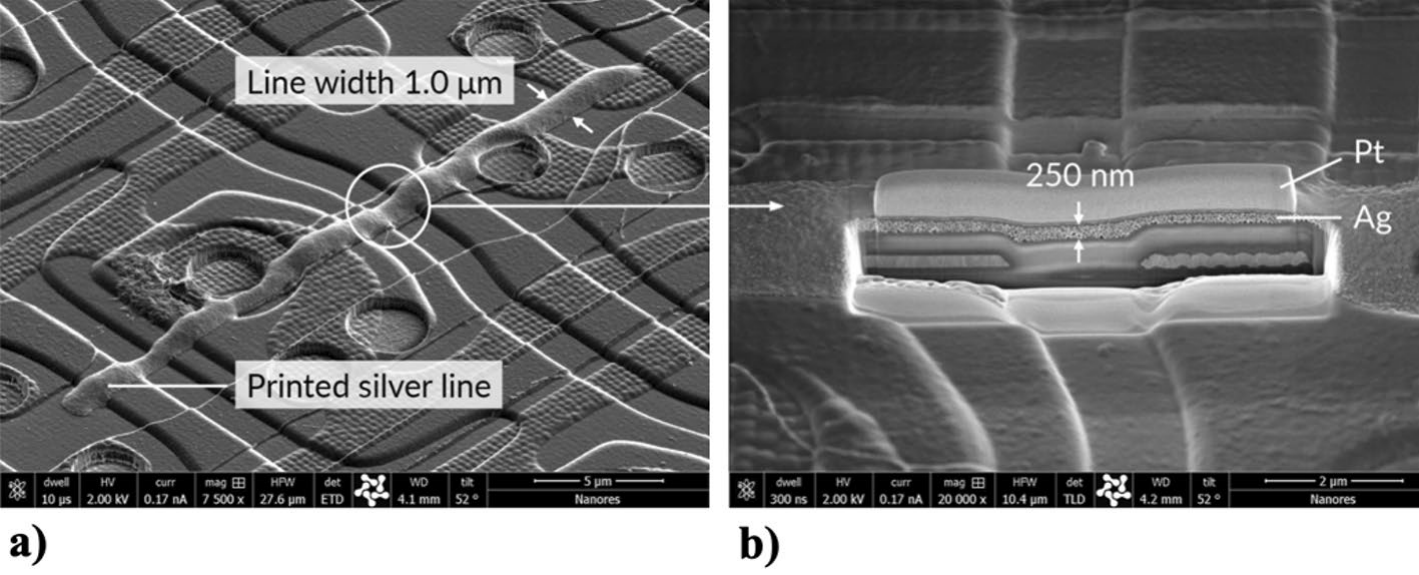
(**a**) Silver line with a width of 1 µm and length of 20 µm printed on an OLED substrate; (**b**) cross section of the line. Platinum layer is for imaging purposes.

Finally, the UPD features presented so far allow to demonstrate an electrical connection printed on a real OLED substrate. In Fig. 15a we show a silver line with a width of 1 µm and length of 20 µm printed on an OLED substrate, whereas in Fig. 15b we demonstrate a cross section of the line. Platinum layer is for imaging purposes.

### Array of source/drain structures for printed flat panel display

**Figure 16:**
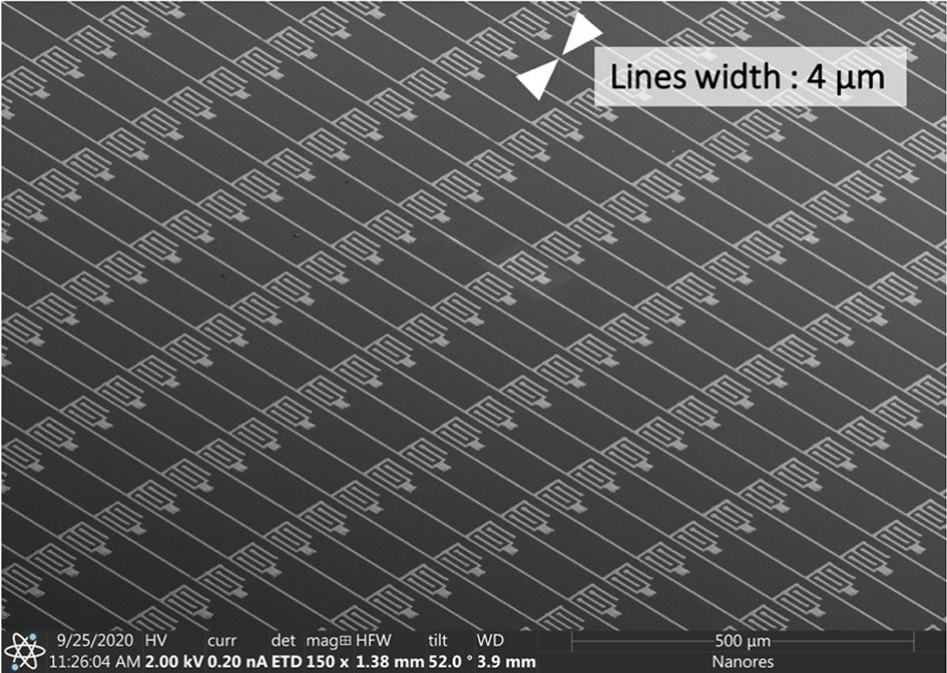
Demonstration of the capabilities of the UPD technology for mass production. The figure shows 7500 printed segments for a thin-film transistor array with the line width of 4 μm. The key feature of UPD in this case is not only the line width, but also the ability to reduce the interline distance to single micrometers and below.

In Fig. 16 we demonstrate the capabilities of the UPD technology for mass production. The figure shows part of the sample of 7500 printed segments for transistors with the line width of 4 μm. The key feature of UPD in this case is not only the line width, but also the ability to reduce the interline distance to single micrometers. Moreover, the shape to be printed can be defined arbitrarily, which supports lean manufacturing.

### LED matrix powered using printed silver lines

**Figure 17:**
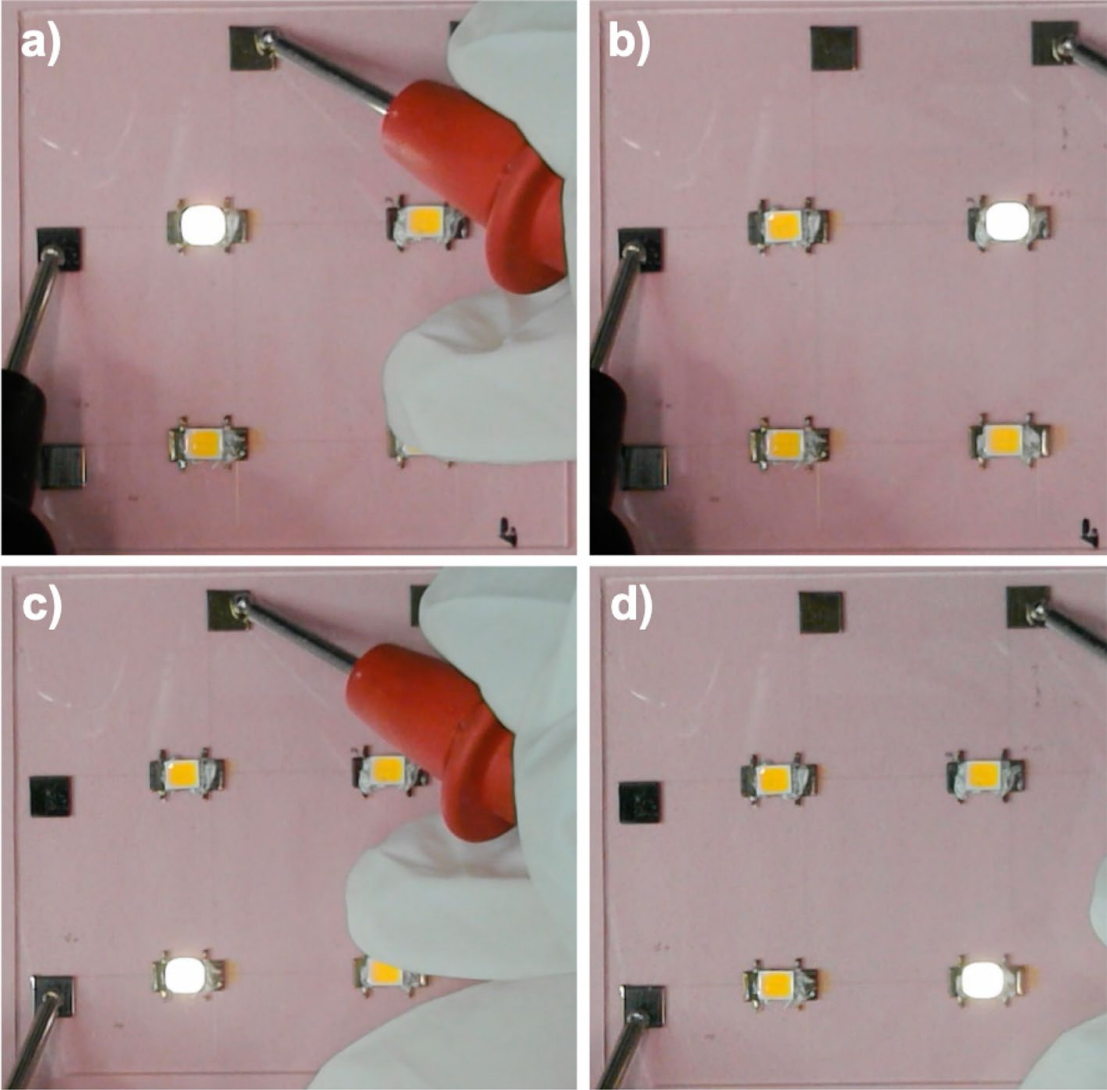
2 × 2 matrix consisting of Surface Mounted Device (SMD) LEDs and powered using printed silver lines made from CL85 paste. The matrix size is 5 × 5 cm and the line width is around 16 µm. Mounting the LEDs and measurements: courtesy of dr. Selen Solak from Humboldt-Univeristät.

As a final case, we give an example of a 2 × 2 matrix consisting of Surface Mounted Device (SMD) LEDs and powered using printed silver lines made from CL85 paste. The sample was fabricated as follows: First, we printed two horizontal conductive tracks. Then, we deposited four segments of insulating material (polyimide ink with the viscosity of 4000 cP) where the horizontal and vertical conductive tracks were planned to intersect to avoid short circuit. Finally, we deposited vertical conductive tracks and pads for SMD LEDs. In this case, we used the UPD approach to deposit both conductive and insulting materials. The resulting matrix size is 5 × 5 cm and the line width is around 16 µm. In Fig. 17 we show lighting up of the diodes one by one.

### Durability of the printed structures

Durability and long-term stability of the fabricated structures are important factors for industrial applications. In this section we discuss an impact of various external parameters on the printed structures: long-term stability, adhesion of the printed structures to the substrate, resistance to high temperatures, multiple folding, impact of humidity, and UV exposure.

For long-term stability, the relative increase of electrical resistance of silver lines with the width of 10 µm after two weeks is around 6.5%. The samples were kept in vacuum, but we used no other protection, like passivation/encapsulation layer. In practical cases, the printed structures would be embedded in microelectronic systems with much higher level of protection. Therefore, we would assume that the relative resistance increase of 6.5% in two weeks after printing is the worst-case scenario.

**Figure 18:**
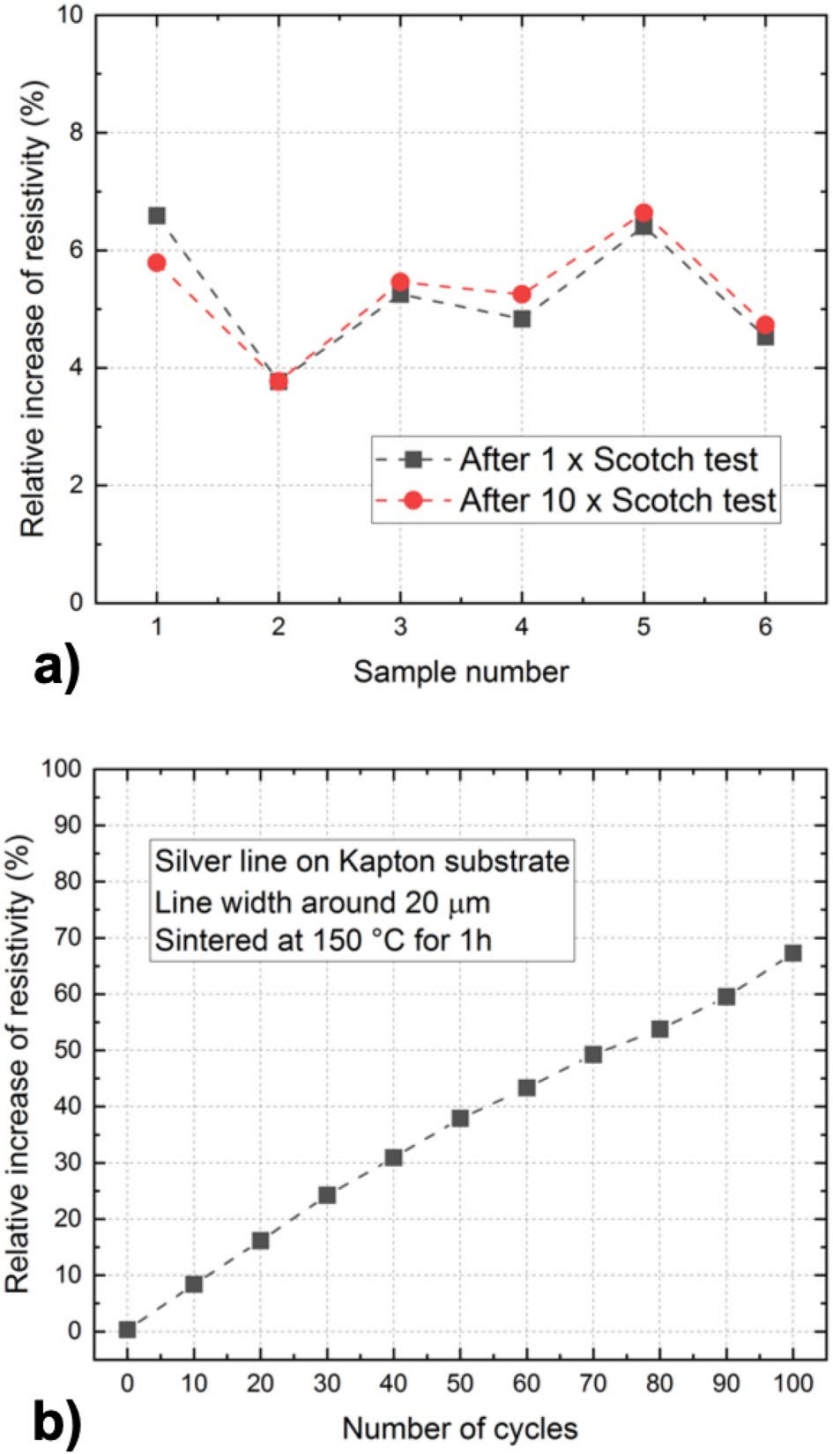
(**a**) Adhesion test of the printed structures: relative increase of resistivity for 5 µm wide silver line after performing 1 × Scotch-tape test and 10 × Scotch-tape test. (**b**) Multiple folding test: relative increase of resistivity as a function of the number of folding cycles.

To test the adhesion of the printed structures, we prepared a number of samples and measured the resistivity before the adhesion test, after a single Scotch-tape test, and after ten Scotch-tape tests. The relative increase of electrical resistance for 5 µm wide silver lines after performing standard 10 × Scotch test is between 4 to 7%. The results are shown in Fig. 18a.

We also performed multiple folding tests of silver lines printed on Kapton substrate, up to 100 bending cycles. The line width is in the range from 21 to 23 µm, and the structures were sintered at 150 °C. The results are shown in Fig. 18b.

When it comes to the impact of high temperature on the printed structures, the practical limitation is the substrate. Besides photonic sintering, we use thermal sintering to sinter the printed structures. The highest temperature we achieved during the thermal sintering of the structures printed on glass is 350 °C (e.g., lines with the width of 5 µm were placed in the oven for 10 min at 350 °C). The printed structures not only survived such high temperature, but we also achieved the best electrical conductivity. However, in the case of structures printed on a PET foil, the maximum sintering temperature is 120 °C^35^, which is the limitation related to the substrate. For PEN foil, the maximum temperature we used was 269 °C.

Humidity may be detrimental to the printed structures. However, printed conductive structures are usually protected from environmental factors by coatings and encapsulation^36,37^. Using the UPD method and the materials discussed above, one can coat printed metallic structures with SU8, deposited using the same printing system.

Finally, regarding UV exposure, we use UV lamp to harden SU8 (wavelength is 365 nm, maximal irradiance is 500 µW/cm^2^) and in many cases printed metallic structures are exposed to UV light as well. E.g., part of the printed line is covered with SU8, so that we can print another line on top, perpendicular to the first one, to achieve an intersection and avoid short-circuit. For the parameters of the UV light mentioned above, we have not observed any detrimental effect of UV light on the printed structures.

## Conclusions

In this contribution we demonstrated the Ultra Precise Deposition (UPD) technology, a versatile platform for depositing conductive and non-conductive materials at micrometer scale. We discussed the working principle of this approach, the means to control the process, the printing system, and we gave a number of examples demonstrating the capabilities of this approach. By these examples we wanted to address common challenges in modern flexible and printed electronics: high-resolution printing of conductive structures, printing on flexible substrates, printing on substrates with pre-existing features including steps much higher than the line width, and deposition of dots. We also gave more specific application examples: array of source/drain structures for printed flat panel display, OLED defect repair (an example of printing on a complex substrate), and LED matrix powered by printed silver lines. In the last example we deposited both conductive and non-conductive materials, which may be useful for all-printed devices. For conductive structures, we achieved the electrical conductivity up to around 40% of the bulk value is currently a record conductivity for printed structures with feature size below 10 µm. In the additive manufacturing landscape, UPD is situated between standard 2D printing and stand-alone 3D architectures. Thanks to the features of the UPD technology, we argue that this approach can become an indispensable element of modern production lines of flexible printed electronics, contributing to fast, reliable, and cost-effective fabrication.

## Data Availability

The datasets used and/or analysed during the current study available from the corresponding author on reasonable request.
